# Characterization and functional biology of the soybean aleurone layer

**DOI:** 10.1186/s12870-018-1579-8

**Published:** 2018-12-13

**Authors:** Monica A. Schmidt, Eliot M. Herman

**Affiliations:** School of Plant Sciences/BIO5 Institute/University of Arizona, 1657 E. Helen St, Tucson, AZ 85721 USA

**Keywords:** Aleurone, Transient expression, Soybean, Seed-specific, Sub-cellular localization

## Abstract

**Background:**

Soybean is a globally important oil seed crop. Both the high protein and oil content of soybean seeds make this crop a lucrative commodity. As in higher eukaryotic species with available genomes, the functional annotation of most of soybean’s genes still remains to be investigated. A major hurdle in the functional genomics of soybean is a rapid method to test gene constructs before embarking on stable transformation experiments.

**Results:**

In this paper we describe the morphology and composition of the persistent single-cell aleurone layer that derives from the endosperm of developing soybean seeds. Its composition compared to cotyledonary tissue indicates the aleurone layer plays a role in both abiotic and biotic stress. The potential utility as the aleurone layer as a transient expression system in soybean was shown. As a near transparent single-cell layer it can be used as a transient expression system to study transgene expression and inter- and intra-cellular targeting as it is amenable to microscopic techniques.

**Conclusion:**

The transparent single cell aleurone layer was shown to be compositionally comparable to cotyledonary tissue in soybean with an enrichment in oxidative response proteins and shown to be a potential transient expression platform.

**Electronic supplementary material:**

The online version of this article (10.1186/s12870-018-1579-8) contains supplementary material, which is available to authorized users.

## Background

Soybean is one of the world’s most important crops due to the high content of both oil and protein in its seed. There was over 340 million metric tons of soybean globally grown in 2016 [1]. Last year, in the US alone there was 33 million hectares of soybean that were harvested and subsequently sold for a total of almost $41B [1] with the value coming from its seed composition: typically, 38% protein and 18% oil. Soybean accounts for 56% of the worldwide oilseed production [1]. Nearly 85% of the annual soybean protein meal produced globally is used as animal feed [1]. For these reasons, the modification of seed-specific output traits of soybean is an increasing focus within the agricultural biotechnology community.

Although the sequence of the soybean genome has been determined [2], functional genomics for a large fraction of its genes has yet to be elucidated. The ultimate determination of gene function is often considered to be the endogenous over- or under-expression of a candidate gene. The transformation and regeneration of most dicotyledonous crop plants, especially soybean, is a laborious, costly and detailed process that is routine only in a few academic laboratories. Currently there are two methodologies, both with average efficiencies around 5%, to produce stable transgenic soybean plants: *Agrobacterium*-mediated transformation using organogenesis regeneration [3, 4] and biolistic transformation using embryogenesis regeneration [5, 6]. For the modification of seed traits in soybean, both transformation methods are tedious and laborious with the *Agrobacterium* method requiring nearly a year for the resulting transgenic plants to mature and set seed before an inserted trait can be assessed [7]. The biolistic/embryogenesis method is more rapid, but still lengthy, for the assessment of seed traits in soybean as mature cotyledonary embryos that are produced after about 6 months of the tissue culture/transformation process can be used as an indicator of seed traits [8–10]. Considering the substantial time and financial investment incurred with either soybean transformation methodologies, efforts to increase the likelihood of obtaining the desired seed phenotype are critically needed before embarking on a stable soybean transformation project. A quick reliable method to assess seed-specific constructs would aid in the efficiency of the determination of gene function and alteration of important traits in the globally important seed crop.

Transient expression assays or model plant transformation systems are used by soybean biotechnologists to forecast if their gene construct will perform as predicted before engineering that construct into soybean [11–13]. These prototypical systems largely involve the use of model plants, such as *Arabidopsis thaliana* [14], *Camelina sativa* [15] or *Nicotiana tabacum* BY-2 [16] cells. Many pitfalls exist when using heterologous systems to assess gene function. For instance, BY-2 cells are non-differentiated cells, so only constitutive regulatory elements can be tested with this tissue. Furthermore, BY-2 cells do not possess seed-specific metabolic pathways and biochemical constituents, many of which are precursors or co-synthesized yielding key seed traits. *Arabidopsis* and *Camelina* plants may be used to assess seed traits but the seeds of *Brassica* species lack some of biochemical pathways and accumulate different types and distributions of storage substances that are present in a soybean seed making it difficult to assess if a soybean seed construct will function correctly in these species. Although efficient and readily employed protocols are in place for *Brassica* model plant transformation systems, it still takes months to generate homozygous transgenic seeds to assess phenotypes for an introduced gene. Well-characterized transient expression systems in plants include the use of onion epidermal peels [17], protoplasts transfections [18] and *Agrobacterium*-leaf infiltration [19]. These systems all have the distinct advantage of being rapid, so results can typically be obtained in under a day, yet they have the same significant disadvantage as none of them are seeds and hence are lacking the necessary constituents to assess an inserted or modified seed trait or seed-specific regulatory elements. There is a need for a rapid protocol to test seed-specific traits in soybean. Here we characterize at the ultrastructure, proteomic and metabolic levels the physiology and composition of soybean’s single-cell aleurone layer that derives from the endosperm and demonstrate that it is functionally similar to maturing cotyledon tissue and, as such, could potentially be used as a platform in a transient expression system.

## Results

### The aleurone is a transparent single-cell layer that contains similar subcellular elements as cotyledonary tissue

**Fig. 1:**
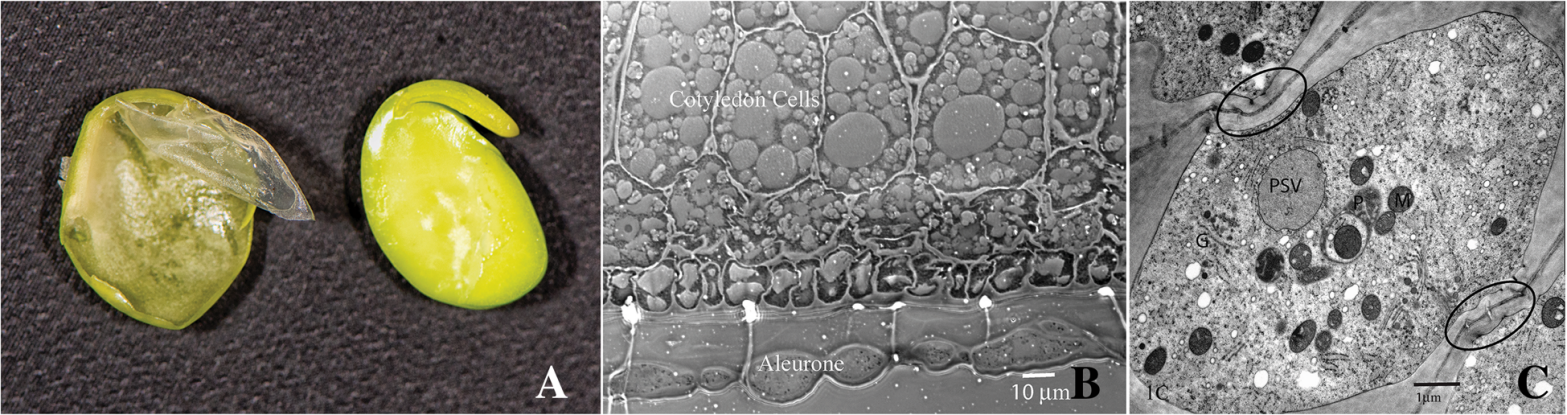
Morphological structure of soybean aleurone layer. **a**) Mature cotyledon showing the isolation of the single-cell aleurone layer from the seed coat. **b**) Light micrograph of a cross-section of a mature soybean cotyledon showing the single cell layer of the aleurone, crushed endosperm and storage parenchyma cells. Bar denotes 10 μm. **c**) Transmission electron micrograph of an aleurone layer isolated from a mature soybean cotyledon. Among the intracellular constituents marked are: PSV protein storage vacuole, P plastid, G golgi, M mitochondria, ER endoplasmic reticulum. Circled areas show the connections between adjacent cells where the plasmadesmata are visible. Bar denotes 1 μm

The morphology of the aleurone layer in soybean was examined at both the gross morphological and subcellular levels building on prior TEM and freeze fracture observations [20]. The single cell layer is potentially an ideal material for high-pressure cryofixation that has the advantage of rapid preservation of subcellular structures and cellular dynamics but is limited by physical dimensions of the subject tissue. Cryofixed excised aleurone was well preserved in the experimental protocols. Ultrathin sections were obtained from freeze-substitute expoxy-embedded aleurones oriented in cross-section that were counter-stained and visualized using a TEM. Figure 1a shows the soybean aleurone layer in relation to a maturing cotyledon and seed coat. A cross-section of the soybean aleurone layer is shown in a light micrograph in Fig. 1b. The aleurone is derived from the endosperm. In maturing soybean seeds the aleurone layer is the single cell layer that is situated between the outer seed coat and inner cotyledonary tissue [20]. The aleurone cells tightly adhere to the cotyledon and axis completely encasing the maturing embryo. The cross-section shows that in between the aleurone and the embryo epidermis layer are dead crushed endosperm cells that are remnants from earlier stages of seed maturation during which the endosperm cells die from programmed cell death leaving only the aleurone. The presence of dead endosperm cells results in a small gap between the remaining aleurone and the epidermis of the cotyledon impeding direct contact of the aleurone and cotyledon. Electron micrograph of aleurone tissue shown in Fig. 1c further defines the tissue’s subcellular structure. Prominent constituent organelles observed in the aleurone electron micrograph include an elaborated endomembrane system, Golgi bodies, cytoplasmic vesicles, oil bodies and protein storage vacuoles typical of seeds, including soybean, that function in the accumulation of reserve proteins and oil.

### Aleurone cells contain a seed-specific proteome

**Fig. 2:**
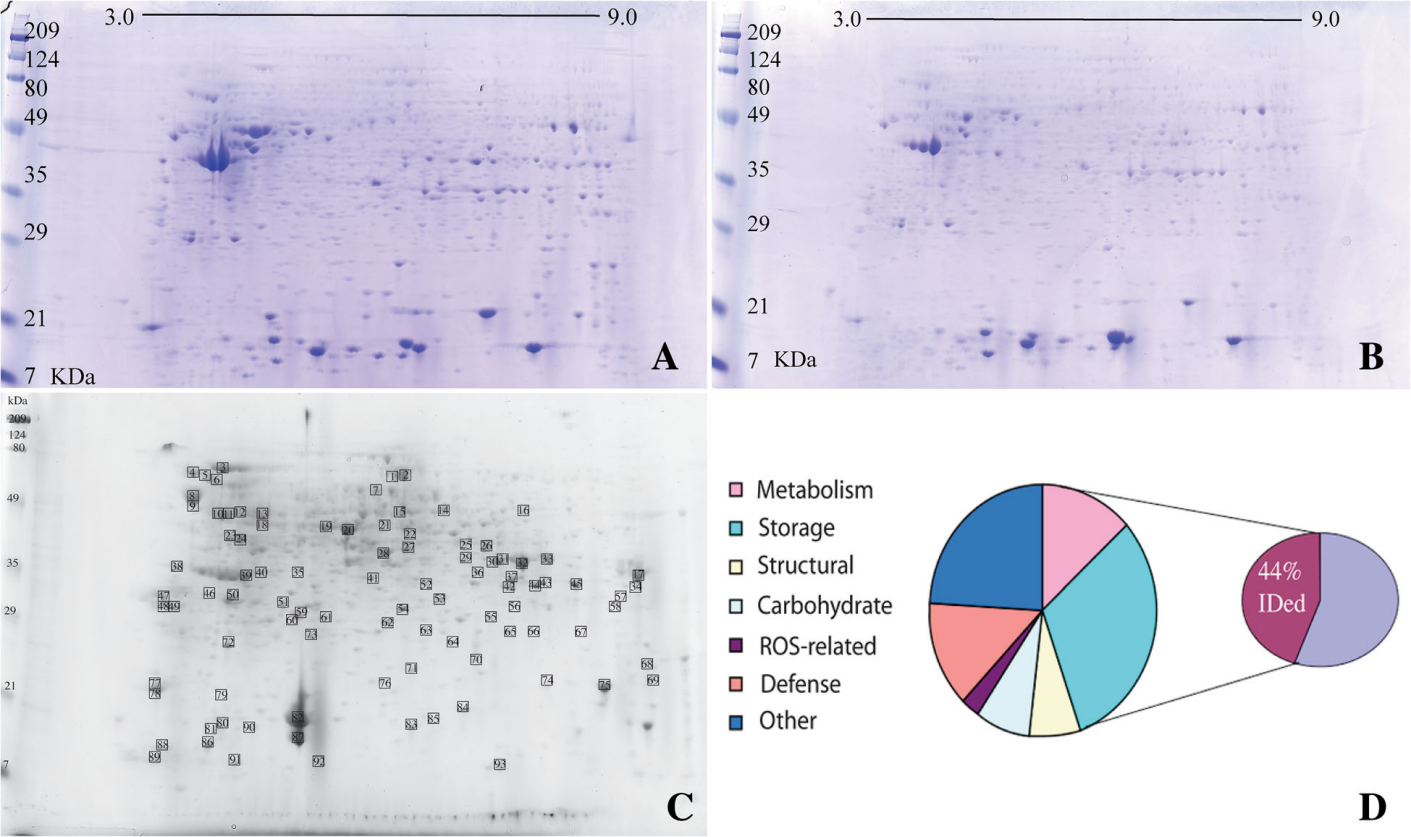
Two-dimensional isoelectric focusing/sodium dodecylsulphate polyacrylamide gel electrophoresis (IEF/SDS-PAGE) analysis of aleurone and mature cotyledon tissue. **a**) Total soluble protein from mature (150 mg) cotyledon tissue. **b**) Total soluble protein from aleurone tissue. **c**) 2-Dimensional SDS-PAGE of aleurone proteome showing 93 proteins isolated and subsequently identified by MS/MS analysis. Spot identification numbers correspond to protein identification numbers listed in Additional file 1: Table S1. **d**) Classification of proteins identified to be present in the aleurone layer of maturing soybean seed. Of the protein spots analyzed for identification, 44% were confidently assayed to a known protein and these proteins were further categorized by their cellular role as involved in metabolism, storage, structural, carbohydrate, oxidative stress, defense or other. Visual comparisons and aleurone protein identifications show the similarity in protein composition of the aleurone to the cotyledonary tissue in soybean

The total soluble proteome of both isolated aleurone layer and cotyledon tissue were analyzed and compared to further characterize the aleurone layer. Two-dimensional IEF/SDS gel analysis of the proteome shows that many of the major proteins found in soybean cotyledons (Fig. 2a) also occur in the aleurone layer (Fig. 2b). Immunoblots of the 2D gels showed comparable immune-reactive proteins in both the cotyledon and aleurone proteome (data not shown). The comparable distribution of both proteins and immune-reactive spots indicates that the proteomes of both the cotyledon and aleurone are dominated by the subunits of the two major storage proteins, β-conglycinin and glycinin, that constitutes nearly 80% a soybean seed’s proteome. Although there are proportional differences in the abundance of various proteins in the aleurone layer compared to the cotyledon tissue indicated by differential protein staining of spots the overall position of the protein spots are in the same location between the gels indicating a large fraction of the proteomes of these tissues overlap. Ninety-three of the more abundant protein spots of the 2-dimensinional gel of the aleurone layer were excised, digested with trypsin, and identified by mass spectroscopy (Fig. 2c). Identification of eighty-six spots were obtained with a high degree of confidence (Additional file 1: Table S1).

The aleurone proteome was compared to prior proteomic analysis from soybean cotyledon/seed tissue, specifically the 111 identified proteins from soybean seeds [9] and 600 identified proteins from 5 maturation stages of soybean seed development [21]. A comparison of the list of proteins identified in the aleurone layer and cotyledon/seed indicates a significant overlap in accumulation of seed-specific proteins including the abundant storage proteins, 7S β-conglycinin and 11S glycinin, as well as soybean anti-nutritional proteins agglutinin and trypsin inhibitor. Although not identified in the present list, a prior study on soybean aleurone identified seed oil body 24 kDa oleosin and the immunodominant soybean seed allergen, P34, using specific antibodies [22]. The relative proportional distribution of the identified aleurone proteins was determined by spot volume analysis of the individual spots and calculated as comprising 44% of the global volume of the proteome displayed on the 2D gel. Identified proteins representing different functional classes were categorized to produce a functional representation of the aleurone proteome. The results of this assessment were calculated and shown in Fig. 2d. One distinct difference between the aleurone and cotyledon proteomes is that proteins with functional assessment in both defense and reactive oxidative species roles are proportionally major constituents of the aleurone proteome in comparison with the previously described proteome of the soybean seed cotyledon [9, 21]. The functional classification of the proteome from developing soybean cotyledons has been previously reported and disease or defense proteins constituted 7% of the identified proteins while no reactive oxygen responsive proteins were detected [21]. Additional file 1: Table S1 shows a list of proteins identified in this study that indicates the aleurone may have additional roles in both biotic and abiotic stress. Of note, one of the most abundant proteins in the aleurone proteome after the storage proteins is a putative disease resistant protein (spots 83 & 87 Fig. 2c and Additional file 1: Table S1). The two disease resistant protein spots resolved as distinct closely positioned spots on the gel yet it remains to be determined if these are isoforms or products of differential processing of a single gene product. Other defense related proteins seen in Fig. 2c include, proteins involved in the scavenging of hydrogen peroxide including thioredoxin (spot 81), 1-cys peroxiredoxin (spot 65), ascorbate peroxidase (spots 60 & 61), glutathione-dependent dehydroascorbate reductase (spot 73) and glutathione peroxidase (spot 76). The presence of reactive oxygen species (ROS) indicates both biotic and abiotic stress responses as well as aging and senescence [23, 24]. Notably, assessing the aleurone layer in comparison with the cotyledon tissue using a dye responsive to the presence of reactive oxidation resulted in enhanced labeling in the aleurone layer providing additional functional confirmation of the assessment derived from proteomics (Additional file 2: Figure S1).

### The aleurone metabolome

**Fig. 3:**
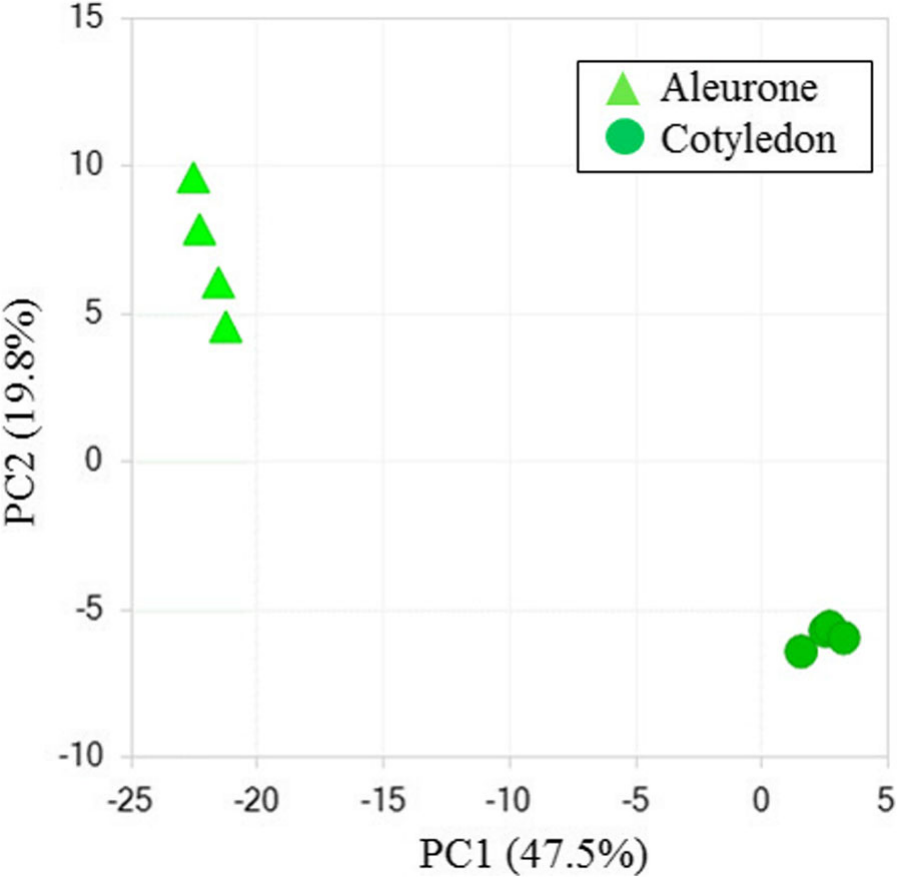
Principal Component analysis comparing the aleurone and cotyledon metabolites. The principal component analysis summarizing the differential results of a non-targeted 452 metabolite analysis. These results illustrate that the metabolic processes of the embryonic aleurone (endosperm) and cotyledon differs

The soybean aleurone is an encasing intermediary between the source maternal seed coat and the maturing zygotic embryo. The source flux could be regulated and distributed by the aleurone to ensure the nutrients are available to support the embryo’s storage substance accumulation. Non-targeted metabolomic analysis of the aleurone’s free-metabolites provides a basis for using its analysis to assess the nutrient influx into the embryo that passes through the endosperm/aleurone early in seed maturation and through the remnant aleurone later in seed maturation after the endosperm’s disintegration. Mid-maturation seeds were harvested to excise both the aleurone and cotyledon that was quench frozen with liquid nitrogen and subsequently processed for non-targeted metabolomic analysis yielding 452 distinct metabolites. The resulting dataset is shown in full in Additional file 3: Table S2 and summarized as a principle component analysis shown in Fig. 3. The metabolomic data indicates that there are some differences in metabolite content characteristic of the aleurone and cotyledon. Within a two-fold cutoff the aleurone is enriched in some glycans, the hydroxyl amino acids threonine and serine, as well as alanine. In contrast the cotyledon is proportionally highly enriched in asparagine – an amino acid that is a primary maternal nitrogen source necessary to support storage protein accumulation. From a botanical perspective the endosperm/aleurone tissue across diverse monocotyledonous and dicotyledonous seeds are proportionally enriched in storage glycans, such as starch and glycomannans, while the embryo, including cotyledons, are often proportionally enriched in storage proteins and oil that is re-iterated in this analysis of the free metabolite comparison.

### Assessment of the aleurone layer as a transient gene expression system

**Fig. 4:**
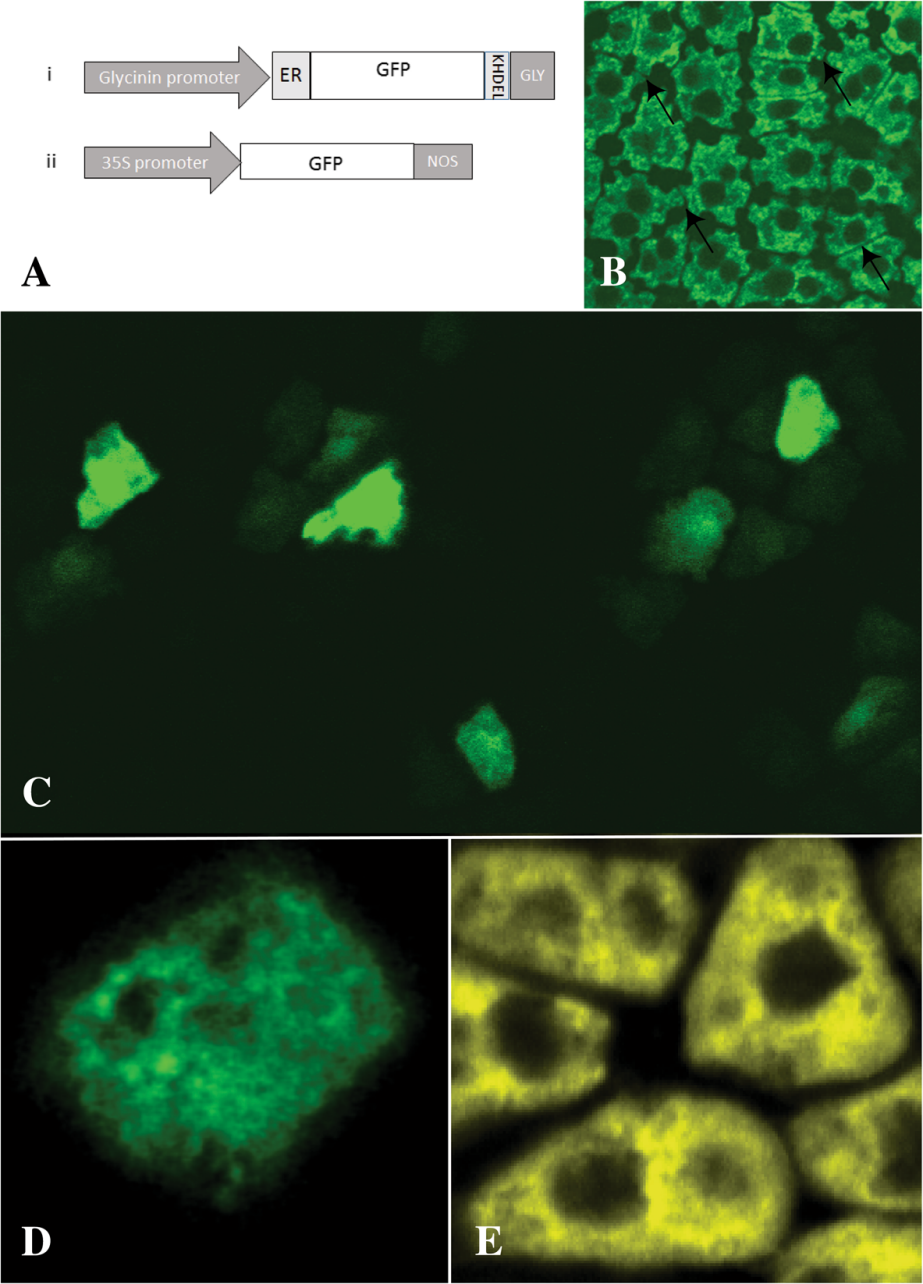
Light micrographs showing transient expression of GFP constructs in aleurone layer. **a**) Graphical representation of constructs used for transient expression assays: i) Seed-specific expression glycinin promoter directing ER-localized GFP ii) Constitutive expression promoter 35S directing the expression of soluble GFP. **b**) GFP fluorescent micrograph of aleurone layer isolated from a previously produced stably transformed soybean plant expressing a seed-specific GFP-ER localized expression cassette. **c**) Soluble enhanced GFP driven by the constitutive promoter transiently expressed in aleurone tissue showing the spread of the GFP fluorescence between neighboring cells that is detectable in as little as 3 hours post-bombardment. **d**) ER-retained GFP driven by a seed promoter is expressed in aleurone cells and able to show the intracellular localization of the fluorescent signal. **e**) Aleurone tissue stained with the internal membrane stain DiOC6 to visualize the ER network for comparison to GFP-ER localized expression seen in stable transformation (**b**) and transient transformation (**d**)

The single cell-thick aleurone layer was assessed for the utility of the visualization of reporter genes. As a transparent tissue it will support high-resolution microscopy as an *ex vivo* living system. The aleurone as an embryonic tissue has many of the same output trait products, in particular storage proteins, that are the commodity meal products of soybean. A previously produced stable soybean transformant expressing a seed-specific green fluorescent protein ER-targeted (GFP-HDEL) construct [25] (Fig. 4a) was used as an indicator control as distribution of the GFP-HDEL expression within the endomembrane system of the aleurone cells (Fig. 4b). This assay provides a morphological control that was used in comparison to evaluate the consequences of the GFP-HDEL transient *ex vivo* expression in excised aleurone. Both a constitutive, 35S, and seed-specific, glycinin, promoters were used to drive the expression of the fluorescent GFP marker in transient biolistic transformation of the aleurone layer (Fig. 4b). The comparison of the GFP expression induced by the constitutive promoter (Fig. 4c) and the seed-specific promoter (Fig. 4d) shows that the soybean aleurone layer is responsive to transient expression system with diverse controlling elements. The seed-specific transient-expressed GFP was observed to distribute to the ER/endomembrane by the open reading framing having both a 5’ ER-signal peptide and a 3’ KHDEL ER retention sequence. The distribution of the transient expression of ER-retained GFP in soybean aleurone cells (Fig. 4d) resulted in a florescent distribution that matched that of the endo-membrane specific dye DiOC6 (Fig. 4e) and to the results from the stable transformation of the aleurone (Fig. 4b). The expression of the cytosolic GFP (Fig. 4c) has distinctly different distribution pattern compared to the ER-retained GFP (Fig. 4d). Cytosolic GFP expression yielded a primary fluorescence locus within transiently transformed cells with significant secondary fluorescence within the adjacent cells. An example shown of three different cells within the same field transiently expressing GFP, with all three cells also showing secondary GFP flow to the adjacent cells. The symplastic flow of GFP from the primary target cell to the adjacent cells is in contrast to the expression of the ER-retained GFP where the GFP synthesized was retained in the primary expressing cells and does not exhibit cell-to-cell flow from the transformed cell to neighboring cells (Fig. 4d). This demonstrates that the transiently transformed cells not only synthesize the transgene product but that the transformed cells are also functionally active and interact with adjacent cells with symplastic flow. From the perspective of the aleurone’s functional morphology as an intermediate tissue between the maternal seed coat source and the zygotic sink the single cell layer aleurone has the functional capacity to act as an interconnected network encasing the embryo that could function to equilibrate distribution of nutritive metabolites assessed by the non-targeted metabolomics (Fig. 3; Additional file 3: Table S2).

## Discussion

Seeds are formed from a double fertilization with the first sperm’s fertilization producing the embryo and the second sperm’s fertilization producing the endosperm. Depending on the plant species, one component of the developing seed matures to be the primary storage tissue, in soybean that is the embryo, whereas in wheat or maize it is the endosperm. In maturing soybean seeds only a single-cell thick aleurone of the endosperm persists as a nearly transparent layer of cells encasing the embryo/cotyledon. The morphology and composition of the cells that constitute the aleurone layer define the function of the persistent aleurone of soybean seeds as an embryonic tissue intermediary between the source maternal seed coat and the sink zygotic embryo but with an intrinsic biology that parallels that of the zygotic embryo that is the agricultural product. The composition analysis of the proteome, metabolome, and structure support the validity of using the soybean aleurone as a proxy for the cotyledon. The biological similarity of the aleurone to the cotyledon, along with the single transparent cell layer morphology, suggested that this tissue could be used as a transient expression assay for seed-specific traits or to assess the functional biology of soybean seeds. The results shown here demonstrate that within a few hours after bombardment, constructs testing seed-specific regulatory elements and subcellular localization could be determined using a fluorescent GFP reporter. This aleurone transient assay is another option for a transient assay of soybean traits. The correct targeting of the GFP transgene product along with its intracellular symplastic flow in its cytosolic form shows that transient aleurone bombardment could be used to study inter- and intracellular processes that is facilitated by the near transparent characteristic of the aleurone that enhances the capacity to use diverse microscopic techniques. Other potential applications of the soybean aleurone include functional evaluation of site-specific mutations (CRISPR) designed to enhance gene regulation function as well as the targeting of transgene products. The rapidity of this assay system allows for a functional mock-up of a transgenic or mutation trait without the lengthy period between the primary and genetic modification and the recovery of primary transformants or mutants.

## Conclusion

The aleurone layer is a biological proxy for soybean’s seed cotyledon. Using morphological analysis, protein, and metabolome datasets we have demonstrated that the single cell layer of the aleurone is comparable to the cotyledon tissue in soybean. It is also enriched with some compounds that indicated it likely plays a functional role in both biotic and abiotic stress resistance. The aleurone as a transparent single cell layer was further shown to be a potentially useful platform for transient gene expression using biolistics and as such would prove useful for functional genomic analysis of genes that affect seed traits within the key seed crop, soybean.

## Methods

### Biological Material

Soybean (*Glycine max* [Merrill] cv Jack) plants from a publically available cultivar were grown in a greenhouse of approximately 25^o^C under 20/4 h day/night photoperiod. Developing cotyledons (150 mg in weight) and aleurone layers were aseptically excised from late maturation (150-200 mg) developing seeds and frozen in liquid nitrogen for later use as starting material for proteome or metabolic analysis.

### Microscopy

Light micrographs were performed with a two-photon excitation Zeiss LSM 510 microscope. For the visualization of GFP, filters with excitation 488 nm and 512 nm emission filters were used. For the visualization of ER membranes, tissue was briefly stained using 1 μg/ml of the ER stain DiOC6 [26] (3,3'-Dihexyloxacarbocyanine Iodide; Fischer Scientific Waltham, MA, USA) and later visualized using filters 482nm for excitation and 504nm for emission. For the visualization of reactive oxygen species (ROS), freshly harvested aleurone tissue was washed with phosphate buffered saline (PBS) pH 7.4 solution for 5 mins and then stained in the dark for with 10 mM 2’7’ dichlorofluorescein diacetate (H2DCF; Invitrogen Molecular Probes; Eugene OR, USA) for 15 mins and rinsed with PBS buffer. Fluorescent microscopy of ROS was taken using a two-photon excitation Zeiss LSM 510 microscope with the parameters of a 488 nm excitation wavelength and detection 500-550 nm bandpass filter. The presence of oxidative stress is seen as green fluorescence under these conditions.

Electron micrographs were performed by initially cryofixing samples with a Balzer’s high-pressure device (Bal-Tech, Principality of Liechtenstein), freeze substituting with acetone-osmium tetraoxide and embedded in epon plastic. Ultrathin sections were stained with both saturated aqueous uranyl acetate and lead citrate (33 mg/mL) prior to observation using a LEO 912AB transmission electron microscope. Images were captured using 2k x 2k charge-coupled device (CCD) camera operated in the montage mode.

### Protein Extraction and 2-Dimensional Analysis

Total protein was extracted from many isolated and subsequently pooled aleurone single cell layers harvested from mature (~150 mg) developing soybean cotyledons and from the cotyledons themselves by a modified phenol method [21, 25]. Samples were ground in liquid nitrogen and resuspended in 0.5mL of Tris-buffered phenol (pH 8.8) and 0.5 mL of extraction buffer (0.1M Tris-HCl pH8.8, 10 mM EDTA, 4% (v/v) 2-mercaptoethanol and 0.9 M sucrose) [27]. Protein pellets were washed as previously described [21] and resuspended in DeStreak rehydration solution (GE Healthcare, PA, USA). Protein was quantitated by Bradford assay. Approximately 150 mg of protein from both aleurone and cotyledon tissue were separately hydrated onto an 11-cm immobilized pH gradient (IPG) gel strip (pH 3-10 nonlinear) (BioRad, CA, USA). Isoelectric focusing was performed for a total of 40 kVh using a Protean IEF Cell (BioRad, CA, USA) and then separated by molecular weight by running the second dimension SDS-PAGE gradient gel (8-16% linear) for 15 mins at 60 V and then 200 V for 1 h. Gels were stained overnight in 0.1% (w/v) Coomassie Brilliant Blue in 40% (v/v) methanol and 10% (v/v) acetic acid followed by many de-staining washes for a total of 3 hours with the same solution void of the stain.

Aleurone two dimensional gels were run in triplicate. Image analysis was performed on Phoretix 2D software (Nonlinear Dynamics Ltd, Newcastle, UK). Protein spots of interest on the aleurone gels were excised and in-gel digestion of the proteins preformed as [28] with a 37^o^C 10 h incubation in 50 mM ammonium bicarbonate containing 6 μg/ml modified trypsin (Promega, Madison WI, USA). Peptides were subsequently extracted by 1% (v/v) formic acid/2% (v/v) acetonitrile and then with 60% acetonitrile. The peptides were then dried and resuspended in 1% (v/v) formic acid/2% (v/v) acetonitrile and then performed ZipTip (Millipore; Ramona CA, USA) according to the manufacturer’s instructions. A QSTAR XL (Applied Biosystems; Foster City CA, USA) hybrid quadrupole TOF MS/MS system was used for peptide sequence data attainment. The peptide electrospray tandem mass spectra were processed using Analyst QS software (ABI) and searched against NCBI EST database using Mascot with the following parameters: oxidation of methionine and carbamindomethylation of cysteine. Positive identifications were made considering the following parameters: (a) number of peptide sequences; (b) protein sequence coverage; (c) total Mascot score; (d) quality of fragmentation data where by a positive is considered to have at least six consecutive amino acids in the full-length y-ion series.

### Metabolomics

Aleurone tissue was collected from fresh maturing soybean cotyledons and flash frozen in liquid nitrogen and used to determine metabolite profile. Three technical replicates were used and profiling was performed by Metabolon Inc. (Durham, NC, USA). Details of liquid chromatography/mass spectrometry (LC/MS) [28] and gas chromatography/mass spectroscopy (GC/MS) [29] were as previously described. Samples were initially extracted in 80% methanol/20% water (v/v) and subsequently divided into three fractions: one was analyzed by LC/MS optimized for positive ionization; the second LC/MS optimized for negative ionization and the third for GC/MS analysis following derivation. Blank samples allowed for the identification of artificial peaks. Raw data files were examined, true chemical peaks were identified using Metabolon’s proprietary peak integration software and by the comparison to library entities of known purified standards or recurrent unknown entities [30, 31]. A combination of chromatographic properties and mass spectra data gave an indication of a chemical match to a specific compound or an isobaric entity [32, 33]. Data were analyzed using Array Studio statistical software (Omic Soft Corporation, Cary NC, USA). Raw integrated peak ion counts were scaled to the median value for each compound and then converted to log scale.

### Plasmid Constructs

The visual marker gene, GFP (green fluorescent protein) was commercially obtained from Clontech^TM^ (Mountain View CA, USA). Both a N-terminal 120 nucleic acid endoplasmic reticulum (ER) signal sequence from *Arabidopsis* chitinase gene and a C-terminal ER khdel retention tag, both as previously described [25, 34, 35] were added to the GFP and the open reading frame was placed between the major soybean storage protein glycinin regulatory elements [25, 34–36]. A construct containing a GFP open-reading frame with no subcellular targeting and under the expression of the strong constitutive cauliflower mosaic virus (35S) promoter was obtained (Addgene plasmid #80127).

### Particle Bombardment of Aleurone Tissue

Maturing soybean cotyledons (150-200 mg in weight) were freshly harvested from greenhouse-grown plants. To harvest the aleurone layer, cotyledons were sliced into half using a razor blade and soaked in a petri dish of water. The aleurone layer was located between the seed coat and cotyledon tissue and peeled back using shape-ended tweezers. Each aleurone tissue was unfolded and placed as single sheets on petri dishes in minimal Basal MS media. The plasmids for bombardment were grown overnight in *E.coli* DH5α cells using a maxi-prep plasmid isolation kit (Qiagen, Hilden, Germany). Gold particles, 0.6 μm in diameter, were used to precipitate approximately 25 μg plasmid DNA using calcium chloride and spermidine. Bombardment was carried out using PDS-1000 (BioRad, CA, USA) with 1100 psi helium pressure. Aleurone bombarded tissue was kept on moist sterile filter paper in a petri dish to maintain humid conditions. The endosperm sheets are incubated in the dark for at least 2-7 h before any analysis was conducted on transient expression.

## Additional files

Additional file 1: **Table S1.** The identification of proteins from 2D gels of aleurone soybean tissue. The columns on the table indicate by number the spot on the gel from Fig. 2, the number of peptides, sequence coverage and Mascot score. Last column denotes if that particular protein had been identified in soybean cotyledon/seed in prior research by: 1) [9] 2) [21]; 3) both [9, 21]; 4) neither [9, 21]. (DOCX 21 kb)

Additional file 2: **Figure S1.** Fluorescent light micrograph for the detection of reactive oxygen species (ROS) in soybean’s aleurone layer. Cross-section of a mature developing soybean showing an outer layer of aleurone alongside the cotyledon tissue both stained with dichlorofluorscein diacetate. The high level of fluorescence detected in the aleurone layer, compared to the cotyledon tissue, indicates an abundance of oxidative activity in the aleurone layer. Bar = 25 μm. (TIF 14966 kb)

Additional file 3: **Table S2.** The metabolites detected in the aleurone and cotyledonary soybean tissue. Shown is metabolic profiling of the cotyledon and aleurone tissues. Major metabolites detected in the cotyledon and aleurone tissues were grouped into those metabolites with higher levels in the aleurone, most sugars for instance, and those with higher abundance in the cotyledon, amino acids and asparagine. (XLSX 2135 kb)

## Abbreviations

DiOC6: 3,3'-Dihexyloxacarbocyanine Iodide
ER: Endoplasmic reticulum
G: Golgi
GFP: Green fluorescent protein
GFP-HDEL: Green fluorescent protein retained to the endoplasmic reticulum
IEF/SDS: Isoelectric focusing sodium dodecyl sulfate
M: Mitochondria
P: Plastid
PSV: Protein storage vacuole
ROS: Reactive oxygen species
