# Supplementary material for: Characterization and functional biology of the soybean aleurone layer
Source: BMC Plant Biol. 2018 Dec 13;18:354. doi: 10.1186/s12870-018-1579-8 (PMC6293662; doi:10.1186/s12870-018-1579-8)
Supplement: Supplementary file 1 — Table S1. The identification of proteins from 2D gels of aleurone soybean tissue. The columns on the table indicate by number the spot on the gel from Fig. 2, the number of peptides, sequence coverage and Mascot score. Last column denotes if that particular protein had been identified in soybean cotyledon/seed in prior research by: 1) [9] 2) [21]; 3) both [9, 21]; 4) neither [9, 21]. (DOCX 21 kb) [file 12870_2018_1579_MOESM1_ESM.docx]

| **Spot ID** | **Kda** | **pI** | **Accession** | **Protein name** | **% Coverage** | **Mascot** | **peptides** | **Prior ID** |
| --- | --- | --- | --- | --- | --- | --- | --- | --- |
| 1 | 19.7 | 5 | gi 33325957 | methionine synthase | 36 | 262 | 7 | 2 |
| 2 | 13 | 5.29 | gi 29469054 | sucrose-binding protein 2 | 81 | 342 | 8 | 3 |
| 3 | 24.5 | 5.47 | gi 32328882 | prepro beta-conglycinin alpha' subunit | 59 | 269 | 13 | 3 |
| 4 | 24.5 | 5.03 | gi 14245736 | beta-conglycinin alpha' subunit | 54 | 537 | 16 | 3 |
| 5 | 21.8 | 5.74 | gi 45331285 | 70 kDa heat shock protein | 23 | 145 | 4 | 2 |
| 6 | 21.4 | 8.94 | gi 762844 | heat shock protein cognate 70 | 28 | 146 | 4 | 2 |
| 7 | NA | NA | NA | not identified | NA | NA | NA | NA |
| 8 | 29.2 | 5.28 | gi 99992 | protein disulfide isomerase | 56 | 470 | 12 | 4 |
| 9 | 29.2 | 5.28 | gi 99992 | protein disulfide isomerase | 59 | 556 | 16 | 4 |
| 10 | 20.2 | 5.76 | gi 50932681 | putatative ATP synthase beta chain | 47 | 319 | 9 | 4 |
|  | 22.2 | 5.71 | gi 9294108 | actin | 29 | 137 | 7 | 4 |
|  | 29.2 | 5.28 | gi 99992 | protein disulfide isomerase | 52 | 448 | 12 | 4 |
| 11 | 21.4 | 7.14 | gi 62122631 | beta-amylase | 39 | 156 | 5 | 4 |
| 12 | 21.8 | 4.91 | gi 3753592 | putative glucan 1,3-beta glucanase | 23 | 132 | 4 | 2 |
| 13 | 21.8 | 4.91 | gi 37533592 | putative glucan 1,3-beta glucanase | 35 | 202 | 6 | 2 |
| 14 | 32.7 | 5.05 | gi 6934188 | actin | 36 | 285 | 9 | 4 |
| 15 | 20.3 | 9.77 | gi 114411 | Mit. a ATP synthetase | 27 | 89 | 5 | 4 |
| 16 | 21.8 | 9.51 | gi 17865455 | catalase 3 | 12 | 106 | 4 | 2 |
| 17 | 23.3 | 6.92 | gi 295810 | elongation factor 1-alpha | 33 | 208 | 10 | 1 |
| 18 | 16.8 | 8.9 | gi 25809054 | DEAD box RNA helicase | 29 | 91 | 4 | 4 |
| 19 | 32.1 | 9.01 | gi 15425637 | beta-conglycinin beta subunit | 43 | 474 | 15 | 1 |
| 20 | 19.7 | 5.04 | gi 256427 | CG4 beta-conglycinin subunit | 35 | 262 | 12 | 1 |
| 21 | 20.1 | 9.59 | gi 7385201 | beta-ketoacyl ACP snythetase | 39 | 154 | 6 | 2 |
| 22 | 29.1 | 5.85 | gi 38636526 | formate dehydrogenase, mitochondrial | 30 | 177 | 6 | 1 |
| 23 | 29.7 | 5.48 | gi 21593328 | actin 11 | 35 | 186 | 6 | 4 |
| 24 | 32.7 | 5.05 | gi 6934188 | actin | 43 | 352 | 13 | 4 |
| 25 | 28.8 | 7.08 | gi 51536124 | formate dehydrogenase, mitochondrial | 35 | 367 | 10 | 1 |
| 26 | 28.3 | 8.7 | gi 22597178 | alchohol dehydrogenase 1 | 25 | 165 | 8 | 2 |
| 27 | 19 | 4.76 | gi 1161602 | phosphoglycerate kinase | 36 | 125 | 5 | 4 |
| 28 | 19.9 | 5.84 | gi 452767 | alchohol dehydrogenase 1 | 38 | 319 | 9 | 2 |
| 29 | 21.7 | 9.52 | gi 62816190 | glyceraldehyde-3-phosphate dehydrogenase | 42 | 246 | 10 | 1 |
| 30 | 18.6 | 6.82 | gi 4539543 | glyceraldehyde-3-phosphate dehydrogenase | 41 | 185 | 7 | 1 |
| 31 | 20.6 | 7.67 | gi 4539543 | glyceraldehyde-3-phosphate dehydrogenase | 67 | 486 | 16 | 1 |
| 32 | 20.3 | 6.17 | gi 462137 | glyceraldehyde-3-phosphate dehydrogenase | 72 | 406 | 18 | 1 |
| 33 | 21.2 | 6.42 | gi 927505 | fructose 1,6-bisphosphate aldolase | 36 | 295 | 10 | 1 |
| 34 | 20.3 | 9 | gi 23731208 | putative GDSL-motif lipase | 51 | 340 | 14 | 4 |
| d35 | 23.8 | 8.44 | gi 1309256a | glycinin A1aBx | 31 | 167 | 5 | 4 |
| 36 | NA | NA | NA | not identified | NA | NA | NA | NA |
| 37 | 20.1 | 6.49 | gi 9622153 | seed maturation protein PM34 | 26 | 194 | 5 | 2 |
| 38 | 16.5 | 4.25 | gi 255224 | glycinin G4 subunit | 31 | 141 | 4 | 1 |
| 39 | 23.8 | 8.44 | gi 18635 | glycinin G1 subunit | 44 | 169 | 7 | 1 |
| 40 | 13.3 | 8 | gi 225651 | glycinin A1aBx | 18 | 72 | 2 | 4 |
| 41 | 18.9 | 6.72 | gi 5524682 | Ni-binding urease accessory protein | 15 | 94 | 5 | 2 |
| 42 | 23.7 | 8.62 | gi 3176098 | annexin | 53 | 474 | 12 | 1 |
| 43 | 23.7 | 8.62 | gi 3176098 | annexin | 53 | 474 | 12 | 1 |
| 44 | 20.9 | 8.72 | gi 3176098 | annexin | 41 | 500 | 11 | 1 |
| 45 | 29.3 | 7.89 | gi 3176098 | annexin | 41 | 432 | 13 | 1 |
| 46 | 27.8 | 4.84 | gi 50931081 | putative 26S proteasome regulatory subunit | 18 | 140 | 4 | 4 |
| 47 | 27.4 | 4.76 | gi 47755555 | 14-3-3-like protein | 25 | 149 | 5 | 2 |
| 48 | 22.1 | 5.21 | gi 1575725 | SGF14A (14-3-3 isoform) | 50 | 364 | 13 | 2 |
| 49 | 17.2 | 5.38 | gi 47755555 | 14-3-3-like protein | 9 | 72 | 4 | 2 |
| 50 | 20.9 | 5.56 | gi 282898 | lecin precursor | 54 | 348 | 12 | 3 |
| 51 | NA | NA | NA | not identified | NA | NA | NA | NA |
| 52 | 31.8 | 9.15 | gi 7488887 | short chain alcohol dehydrogenase | 29 | 241 | 9 | 4 |
| 53 | 19.8 | 6.13 | gi 57506548 | glucose and ribitol dehydrogenase | 29 | 115 | 5 | 1 |
| 54 | 21.9 | 9.63 | gi 282901 | maturation protein MAT9 | 11 | 70 | 2 | 4 |
| 55 | 22.3 | 6.26 | gi 28950805 | putative embyro-specific protein | 51 | 257 | 13 | 4 |
| 56 | 19.1 | 10.32 | gi 12229936 | proteasome subunit alpha | 25 | 142 | 5 | 4 |
| 57 | 20.3 | 9.08 | gi 38640795 | endo-1,3-glucanase | 31 | 164 | 6 | 2 |
| 58 | 16.6 | 9.4 | gi 4260342 | mitochondrial voltage-dependent porin | 51 | 140 | 5 | 2 |
| 59 | 25.7 | 5.47 | gi 20197253 | putative protein | 30 | 200 | 7 | 4 |
| 60 | 27 | 5.78 | gi 37196685 | cytosolic ascorbate peroxidase 1 | 32 | 198 | 6 | 4 |
| 61 | 24.9 | 5.06 | gi 37196687 | cytosolic ascorbate peroxidase 2 | 32 | 188 | 8 | 4 |
| 62 | 29.5 | 5.89 | gi 48773765 | trisephosphate isomerase | 36 | 277 | 8 | 4 |
| 63 | 20.3 | 6.88 | gi 7488657 | cysteine proteinase inhibitor | 27 | 120 | 5 | 4 |
| 64 | NA | NA | NA | not identified | NA | NA | NA | NA |
| 65 | 21.1 | 8.95 | gi 49618728 | 1-cys peroxiredoxin | 37 | 274 | 9 | 4 |
| 66 | NA | NA | NA | not identified | NA | NA | NA | NA |
| 67 | 24.8 | 8.48 | gi 3421123 | 20S proteasome beta-subunit PBG1 | 14 | 69 | 3 | 4 |
| 68 | 20.1 | 9.64 | gi 806556 | glycinin A5A4B3 subunit | 27 | 279 | 6 | 3 |
| 69 | 20.1 | 9.64 | gi 806556 | glycinin A5A4B3 subunit | 27 | 255 | 4 | 3 |
| 70 | 23.2 | 7 | gi 23306428 | putative strictosidine synthase | 50 | 364 | 13 | 4 |
| 71 | 13 | 6.92 | gi 4803937 | glycine rich protein A.thaliana | 24 | 121 | 3 | 4 |
| 72 | 22.6 | 9.13 | gi 5733686 | maturation protein pPM32 | 13 | 121 | 4 | 2 |
| 73 | 24.4 | 5.68 | gi 21593056 | GSH-dependent dehydroascorbate reductase | 40 | 330 | 9 | 4 |
| 74 | 18.7 | 7.85 | gi 225651 | glycinin A1aBx | 53 | 428 | 14 | 4 |
| 75 | 20.3 | 6.34 | gi 225651 | glycinin A1aBx | 67 | 532 | 15 | 4 |
| 76 | 19.7 | 6.18 | gi 37930463 | glutathionine peroxidase | 54 | 268 | 9 | 4 |
| 77 | 19.2 | 7.56 | gi 39725577 | trypsin inhibitor | 36 | 293 | 9 | 1 |
| 78 | 20.4 | 5.2 | gi 18770 | trypsin inhibitor | 32 | 252 | 9 | 1 |
| 79 | 15 | 5.09 | gi 5726567 | glycine rich RNA-binding protein | 60 | 223 | 8 | 4 |
| 80 | 16.6 | 9.4 | gi 67848430 | mitochondrial voltage-dependent porin | 51 | 140 | 5 | 2 |
| 81 | 16.2 | 8.52 | gi 13624884 | thioredoxin | 22 | 179 | 5 | 4 |
| 82 | 21.9 | 8.05 | gi 28972750 | putative disease response | 20 | 197 | 7 | 4 |
| 83 | 18.2 | 9.15 | gi 1236951 | nucleoside diphosphate kinase | 18 | 68 | 3 | 2 |
| 84 | NA | NA | NA | not identified | NA | NA | NA | NA |
| 85 | 19.5 | 9.43 | gi 1236951 | nucleoside diphosphate kinase | 37 | 353 | 12 | 2 |
| 86 | 25.8 | 7.74 | gi 3377794 | proteasome IOTA subunit | 13 | 87 | 4 | 4 |
| 87 | 21.8 | 8.05 | gi 28973750 | putative disease response | 35 | 234 | 8 | 4 |
| 88 | 18.6 | 6 | gi 4097896 | napin-type 2S albumin | 18 | 82 | 3 | 1 |
| 89 | NA | NA | NA | not identified | NA | NA | NA | NA |
| 90 | 24.7 | 7.82 | gi 26449830 | putuative GDSL-motif lipase/acylhydrolase | 22 | 212 | 5 | 4 |
| 91 | 18.5 | 8.15 | gi 5106775 | ribosomal protein S12 Hordeum | 49 | 192 | 10 | 4 |
| 92 | 22.1 | 5.94 | gi 255224 | glycinin G4 subunit | 35 | 183 | 7 | 1 |
| 93 | 19.9 | 9.38 | gi 21537360 | putative glyoxalase | 3 | 92 | 3 | 4 |
