# Supplementary figures and images for: Characterization and functional biology of the soybean aleurone layer
Source: BMC Plant Biol. 2018 Dec 13;18:354. doi: 10.1186/s12870-018-1579-8 (PMC6293662; doi:10.1186/s12870-018-1579-8)

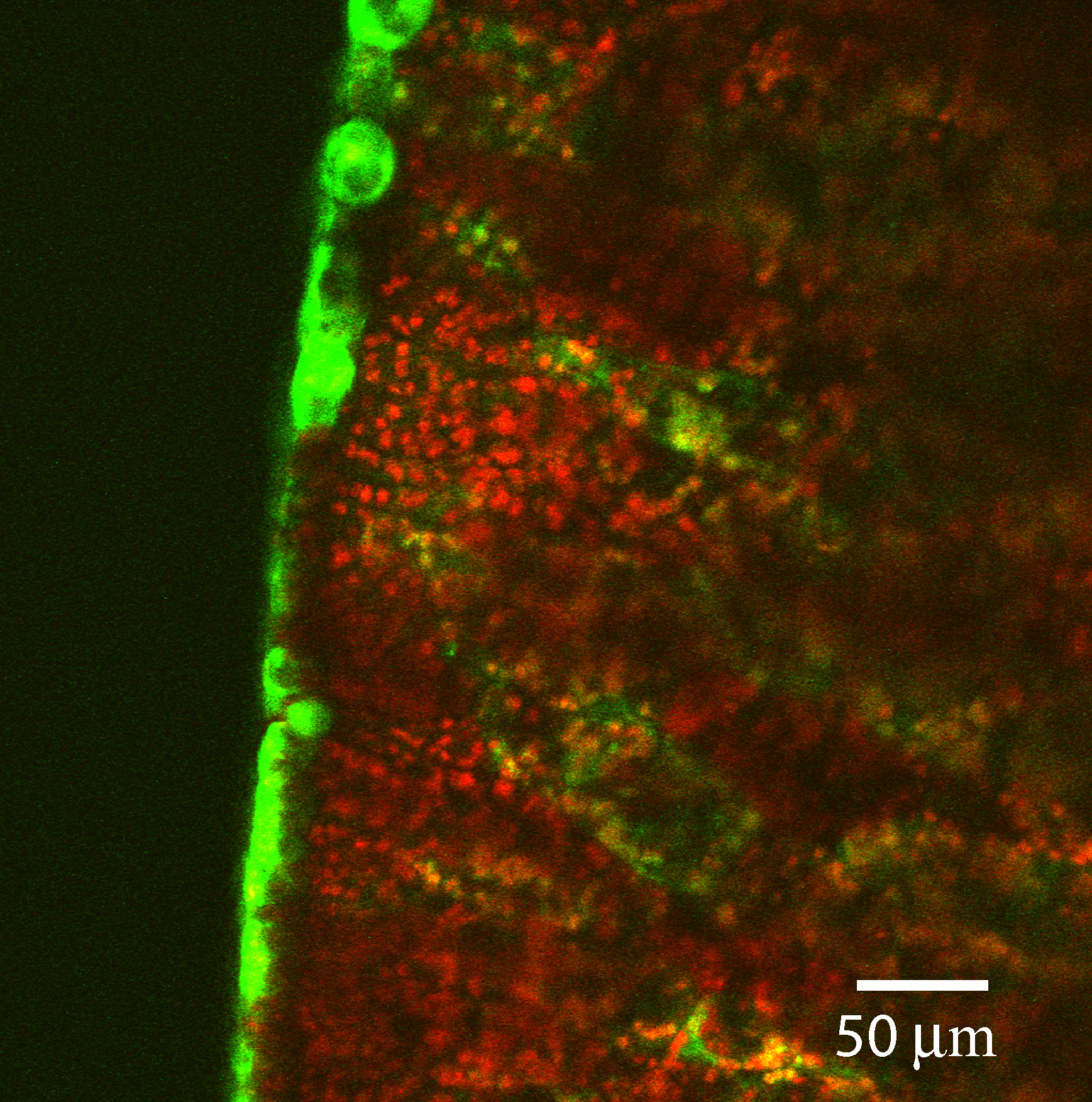

Supplement: Supplementary file 2 — Figure S1. Fluorescent light micrograph for the detection of reactive oxygen species (ROS) in soybean’s aleurone layer. Cross-section of a mature developing soybean showing an outer layer of aleurone alongside the cotyledon tissue both stained with dichlorofluorscein diacetate. The high level of fluorescence detected in the aleurone layer, compared to the cotyledon tissue, indicates an abundance of oxidative activity in the aleurone layer. Bar = 25 μm. (TIF 14966 kb) [file 12870_2018_1579_MOESM2_ESM.tif]
